# Severe Acute Respiratory Syndrome Coronavirus 2 (SARS-CoV-2): Codon Usage and Replicative Fitness

**DOI:** 10.1055/s-0040-1721080

**Published:** 2020-12-02

**Authors:** Darja Kanduc

**Affiliations:** 1 Department of Biosciences, Biotechnologies, and Biopharmaceutics, University of Bari, Bari, Italy

**Keywords:** codon usage, HCoV-OC43, SARS-CoV-2, viral replication fitness

## Abstract

Severe acute respiratory syndrome coronavirus 2 (SARS-CoV-2) codon usage, as shown by the polyprotein coding sequence, shows better translation potential in the human host when compared with human coronavirus OC43 (HCoV-OC43) codon usage. Such translational advantage might facilitate SARS-CoV-2 replication, immunogenicity, and pathogenicity, thus also accounting for the less harmful character of HCoV-OC43 infection.

## Introduction

Severe acute respiratory syndrome coronavirus 2 (SARS-CoV-2) infection causes a respiratory syndrome with altered pulmonary and alveolar function that can evolve into acute respiratory insufficiency and death. 1 Progressive immune-associated injury is a hallmark of SARS, 2 and alteration of the lung functions is possibly due to specific autoimmune cross-reactions 3 4 against alveolar surfactant-related proteins, 5 with a higher titer of antibodies independently associated with a worse clinical classification. 6 In conflict, the human coronavirus OC43 (HCoV-OC43) generally relates to less serious disturbances as common cold. 7 Currently, the molecular determinants and the mechanisms that underlie such a different pathogenic load are unknown.

Based on previous reports 8 9 suggesting that rare host codons can inhibit viral protein expression and favor viral latency, this study investigated the codon usage in HCoV-OC43 and SARS-CoV-2. Specifically, usage of the 61 amino acid (aa) specifying codons was analyzed in the HCoV-OC43 and SARS-CoV-2 polyprotein (aka orf1ab) ORF (open reading frame) and then was compared with the codon usage of the human ORFeome. 10

Main results are reported in Table 1 , which shows the different usage of a set of eight codons, whereas full data for the 61 codons in *Homo sapiens* , HCoV-OC43, SARS-CoV-2, SARS-CoV, and Middle East respiratory syndrome coronavirus (MERS-CoV) are detailed in Supplementary Table S1 (online only).

**Table 1:** Codon usage bias between human ORFeome and polyprotein ORFs from HCoV-OC43 and SARS-CoV-2: a representative analysis of eight codons a b c

Aa	Codon	*Homo sapiens*	HCoV-OC43 d Isolate: 1987	HCoV-OC43 e Isolate: 1990	HCoV-OC43 f Isolate: 2011	SARS-CoV-2 g Isolate: 2019	SARS-CoV-2 h Isolate:2020
Asp	GAT	21.8	54.5	54.5	54.8	35.4	35.5
Phe	TTT	17.6	50.2	49.8	50.6	35.8	35.9
Ile	ATT	16.0	30.7	30.6	31.1	23.8	23.9
Leu	TTG	12.9	32.4	32.4	32.4	16.3	16.5
Asn	AAT	17.0	41.7	41.7	41.9	37.8	37.9
Arg	CGT	4.5	12.0	11.8	11.7	8.3	8.2
Ser	AGT	12.1	25.2	25.7	25.8	16.8	16.7
Tyr	TAT	12.2	45.5	45.5	45.9	29.3	29.2

Abbreviations: HCoV-OC43, human coronavirus OC43; ORF, open reading frame; SARS-CoV-2, severe acute respiratory syndrome coronavirus 2.

a Codon frequency expressed per thousand.

b Codon usage of *Homo sapiens* obtained at www.kazusa.or.jp/codon. ^10^

c Codon usage of polyprotein ORFs obtained at www.geneinfinity.org .

d HCoV-OC43/human/USA/1987; GenBank: KF530083.1; ID = AGT51637.1; taxon:31631.

e HCoV-OC43/human/USA/1990; GenBank: KF530088.1; ID = AGT51687.1; taxon:31631.

f HCoV-OC43/UK/London/2011; GenBank: KU131570.1; ID = AMK59674.1; taxon:31631.

g SARS-CoV-2/Wuhan-Hu-1/2019; GenBank:MN908947.3; ID = QHD43415.1; taxon:2697049.

h SARS-CoV-2/UW239/2020/USA; GenBank: MT251975.1; ID = QIQ68493.1; taxon:2697049.

Table 1 describes the following:

- Eight codons are often used in HCoV-OC43 polyprotein ORF but occur at a lesser extent in the *H. sapiens* ORFeome, with a human-to-viral usage ratio smaller than 1, that is, from the translational point of view, the human-to-viral usage ratio is unfavorable to HCoV-OC43 since the optimal ratio value for HCoV-OC43 polyprotein synthesis in the human host is approximately 1. 8 9 11 12
- The human-to-viral usage ratio remains suboptimal for translational expression in the three HCoV-OC43 isolates collected in 1987, 1990, and 2011, respectively.
- Usage of the eight codons is lower in SARS-CoV-2 polyprotein ORF so that the human-to-viral usage ratio reaches values closer to approximately 1 and is more suitable for the viral polyprotein translation in the human host.

In summary, in the context of CoV polyprotein expression, Table 1 documents that eight codons are more often used in HCoV-OC43 polyprotein ORF than in the human ORFeome and might represent a translational constraint for HCoV-OC43 polyprotein expression, thus limiting HCoV-OC43 replication, diffusion, and pathogenicity, given the essential role of coronavirus polyprotein for generation of viral progeny. 13 14

The possibility of translational block/delay emerges when considering the iteration of the eight suboptimal codons along the HCoV-OC43 polyprotein ORFs. Among many, two examples from the HCoV-OC43 polyprotein (isolate 2011) are the following aa sequences: (1) YDDVNASLFVDYSNL that is coded by a row of codons (given capital) abundant in the viral polyprotein ORF but not in the human ORFeome, TAT-GAT-GAT-gtt-AAT-gct-AGT-TTG-TTT-gtg-GAT-TAT-AGT-AAT-TTG, and (2) IDDHRITSITSDKFDFII that is coded by the viral nucleotide sequence ATT-GAT-GAT-cat-CGT-atc-act-AGT-ATT-act-AGT-GAT-aag-TTT-GAT-TTT-ATT-ATT, where 12 codons out of 15 are suboptimal for translation in the human host. Clearly, the potential to be translated of such HCoV-OC43 coding sequences progressively and severely diminishes along the succession of the suboptimal codons.

To conclude, the data suggest a link between CoV codon usage and CoV pathogenicity in humans. Usage of relatively rare human codons and their clustering along viral sequences can represent a major translational block at the basis of HCoV-OC43 low expression, low immunogenicity, and low pathogenicity. Vice versa, the possibility to be translated is rescued in SARS-CoV-2 polyprotein ORF by the minor usage of rare human codons ( Table 1 ). When compared with HCoV-OC43, the higher translatability of SARS-CoV-2 correlates to a higher viral protein expression and a higher capability of evoking immune responses. Consequently, also, anamnestic immune responses of increased avidity and affinity can arise and lead to autoimmune pathologies in case of repeated exposures to SARS viruses, 15 16 17 18 according to the recently clarified phenomenon of immunologic memory imprinting, also known as Original Antigenic Sin. 19

More in general, the data further support the translational control of viral protein expression as a mechanism by which the human host can silence and tolerize viral invasion. 8 9 Hence, this study warns that microbiology methodologies such as codon optimization, insertion/modification of translational enhancers, or addition of viral vectors *inter alia* , can increase the expression, replicative fitness, diffusion, and pathogenicity of the infectious agents under study, thus altering the finely tuned equilibrium between immunogenicity and immunotolerance. 20 21
